# Sensory Healthcare for People with Dementia in Diverse Care Settings

**DOI:** 10.1002/alz70858_101398

**Published:** 2025-12-25

**Authors:** Iracema Leroi

**Affiliations:** ^1^ Trinity College Institute of Neuroscience, Trinity College Dublin, Dublin, Ireland

## Abstract

The section will build upon the previous presentations within this Clinical Toolbox session and address sensory impairments across various care settings. The session will discuss the prevalence and impact of sensory impairments—hearing and vision loss—in individuals with dementia in care settings, including at home, in receipt of home care packages, and living in long‐term care (LTC) facilities. Between 75% and 90% of residents with dementia (RwD) in LTC experience hearing loss, and approximately 40% face substantial vision loss, rates significantly higher than those observed in home‐dwelling individuals with dementia. Dual sensory impairment exacerbates difficulties in communication, social engagement, and independence, leading to worse quality of life and accelerated cognitive and functional decline.

Despite the high prevalence of sensory impairments, under‐detection and under‐management persist, often due to inadequate sensory health screening, insufficient staff training, and limited resources for addressing sensory needs in cognitively impaired populations. “Sensory unfriendly” environments in LTC, characterized by poor lighting and high noise levels, further compound these challenges. This session will review barriers to effective sensory care, including insufficient use and maintenance of sensory aids, unclear referral pathways, and a lack of training for healthcare professionals.

Using exemplar studies, the session will demonstrate the positive impact of sensory rehabilitation on quality of life. The SENSE‐Cog RCT and related adaptations, including a feasibility pilot of a Sensory Support Intervention (SSI) tailored for LTC settings, will serve as case studies. These interventions show promising short‐term improvements in quality of life, though challenges in achieving long‐term benefits remain. A training package for LTC staff in sensory‐cognitive health will be shared.

Participants will gain insights into the knowledge, awareness, and practices of sensory‐cognitive healthcare among professionals in different settings. By exploring the feasibility and implementation of sensory rehabilitation measures, the session aims to inspire actionable strategies to enhance sensory care for people with dementia, ultimately improving their quality of life and care outcomes.

